# Supplementary material for: Three-Dimensional Organotypic Cultures Reshape the microRNAs Transcriptional Program in Breast Cancer Cells
Source: Cancers (Basel). 2022 May 19;14(10):2490. doi: 10.3390/cancers14102490 (PMC9139376; doi:10.3390/cancers14102490)
Supplement: Supplementary file 1 [file cancers-14-02490-s001.zip › Supplementary Table S1.pdf]

Table S1. List of the 205 miRNAs overexpressed in 3D cultures compared to 2D.

miRNA	Fold Change	p-value	FDR
miR-4417	51.35	1.22E-12	5.46E-09
miR-1290	45.78	5.36E-08	5.82E-06
miR-7641	45.15	1.65E-12	5.46E-09
miR-4449	23.6	4.43E-09	1.32E-06
miR-1246	23.34	8.10E-07	3.61E-05
miR-6780b-5p	22.19	2.64E-06	9.15E-05
miR-3197	21.01	3.71E-08	5.12E-06
miR-3175	20.06	6.27E-08	6.39E-06
miR-1247-3p	19.83	3.20E-08	4.83E-06
miR-6802-5p	18.12	7.17E-06	0.0002
miR-6782-5p	16.42	1.63E-06	6.29E-05
miR-4253	15.68	7.39E-09	1.89E-06
miR-4484	15.62	2.15E-10	2.85E-07
miR-7150	13.92	6.23E-05	0.0012
miR-8089	12.96	2.09E-07	1.31E-05
miR-6716-5p	12.71	4.68E-08	5.64E-06
miR-6824-5p	11.78	7.95E-08	7.08E-06
miR-6787-5p	11.24	1.72E-07	1.18E-05
miR-4327	10.84	7.68E-06	0.0002
miR-1281	10.1	1.54E-07	1.09E-05
miR-6132	10.05	3.72E-05	0.0008
miR-1225-5p	8.77	2.38E-07	1.43E-05
miR-4665-5p	8.47	0.0005	0.0073
miR-5196-5p	8.45	6.22E-08	6.39E-06
miR-4449	7.93	2.22E-06	8.09E-05
miR-4271	7.92	8.68E-09	1.98E-06
miR-4470	7.89	2.15E-05	0.0005
miR-3937	7.88	1.19E-07	9.35E-06
miR-6820-5p	7.33	7.59E-05	0.0014
miR-6879-5p	7.27	1.69E-05	0.0004
miR-939-5p	7.07	1.19E-07	9.35E-06

miR-6870-5p	7.02	2.12E-07	1.31E-05
miR-4649-5p	6.99	1.72E-07	1.18E-05
miR-6126	6.91	2.09E-10	2.85E-07
miR-3651	6.86	8.99E-06	0.0002
miR-4750-5p	6.51	3.35E-05	0.0007
miR-4487	6.4	1.51E-06	5.92E-05
miR-371b-5p	6.31	7.97E-06	0.0002
miR-150-3p	5.97	2.31E-05	0.0005
miR-4758-5p	5.92	2.25E-06	8.09E-05
miR-6891-5p	5.84	3.92E-06	0.0001
miR-3648	5.79	1.27E-06	5.16E-05
miR-6794-5p	5.78	1.64E-05	0.0004
miR-4721	5.78	1.50E-07	1.08E-05
miR-7110-5p	5.62	6.34E-06	0.0002
miR-8063	5.23	0.0003	0.0044
miR-3621	5.23	2.73E-07	1.53E-05
miR-2392	5.12	3.46E-06	0.0001
miR-6861-5p	4.95	1.44E-05	0.0003
miR-6798-5p	4.9	2.45E-06	8.63E-05
miR-1224-5p	4.89	1.89E-06	7.07E-05
miR-6763-5p	4.85	0.0003	0.0047
miR-4632-5p	4.83	2.32E-05	0.0005
miR-572	4.8	3.85E-06	0.0001
miR-3162-5p	4.77	1.54E-06	5.99E-05
miR-3622a-5p	4.77	2.89E-05	0.0006
miR-6790-5p	4.52	4.28E-06	0.0001
miR-7845-5p	4.52	0.0004	0.0059
miR-1202	4.51	3.26E-06	0.0001
miR-6848-5p	4.5	1.90E-07	1.26E-05
miR-5739	4.47	2.78E-05	0.0006
miR-6771-5p	4.42	0.0011	0.0151
miR-619-5p	4.42	1.22E-05	0.0003
miR-3180	4.4	0.0007	0.0095

miR-4467	4.39	1.55E-06	6.03E-05
miR-6749-5p	4.3	4.60E-09	1.32E-06
miR-149-3p	4.15	3.12E-08	4.83E-06
miR-4463	4.1	2.33E-08	4.07E-06
miR-6768-5p	4.03	0.0073	0.0714
miR-1229-5p	4.02	4.23E-05	0.0009
miR-6836-5p	4.02	0.0001	0.0026
miR-6797-5p	3.97	5.58E-08	5.87E-06
miR-4322	3.9	3.83E-06	0.0001
miR-575	3.86	7.82E-05	0.0015
miR-6808-5p	3.83	8.92E-06	0.0002
miR-4433-3p	3.76	1.52E-07	1.09E-05
miR-4433b-3p	3.76	1.46E-07	1.08E-05
miR-3679	3.7	0.0009	0.013
miR-4741	3.7	1.45E-07	1.08E-05
miR-4706	3.66	2.01E-05	0.0005
miR-885-3p	3.64	4.70E-08	5.64E-06
miR-6776-5p	3.57	3.36E-06	0.0001
miR-6756-5p	3.54	6.99E-08	6.89E-06
miR-5195-3p	3.52	0.0012	0.0156
miR-6752-5p	3.52	3.30E-08	4.86E-06
miR-4656	3.49	0.0035	0.0389
miR-4299	3.44	7.23E-08	6.90E-06
miR-6722-3p	3.44	7.66E-09	1.89E-06
miR-6846-5p	3.44	9.80E-06	0.0003
miR-4689	3.43	4.91E-08	5.72E-06
miR-6075	3.42	1.44E-05	0.0003
miR-6723-5p	3.34	0.0018	0.0214
miR-8072	3.29	1.43E-06	5.71E-05
miR-6085	3.27	2.36E-06	8.41E-05
miR-4640-5p	3.25	4.07E-05	0.0008
miR-6889-5p	3.24	0.0002	0.0036
miR-1973	3.19	1.35E-05	0.0003

miR-642b-3p	3.17	0.0002	0.0038
miR-6805-5p	3.16	3.70E-06	0.0001
miR-4281	3.16	4.76E-08	5.64E-06
miR-6803-5p	3.14	5.10E-07	2.51E-05
miR-483-5p	3.12	9.06E-05	0.0017
miR-1254	3.11	1.57E-05	0.0004
miR-6840-3p	3.07	2.72E-06	9.29E-05
miR-3188	3.05	0.0007	0.0097
miR-1343-5p	3.05	8.89E-05	0.0017
miR-3679-5p	3	0.0006	0.0084
miR-6786-5p	2.98	7.06E-08	6.89E-06
miR-135a-3p	2.98	5.97E-05	0.0012
miR-6893-5p	2.97	0.0002	0.0035
miR-937-5p	2.94	3.53E-06	0.0001
miR-30b-5p	2.92	2.01E-07	1.28E-05
miR-6799-5p	2.9	3.28E-06	0.0001
miR-6785-5p	2.89	4.48E-07	2.30E-05
miR-1268a	2.87	6.90E-06	0.0002
miR-1228-5p	2.87	1.49E-06	5.89E-05
miR-1268b	2.86	1.43E-06	5.71E-05
miR-4725-3p	2.85	0.0002	0.004
miR-4462	2.84	5.36E-05	0.0011
miR-3940-5p	2.84	4.09E-07	2.14E-05
miR-6089-1	2.83	2.72E-07	1.53E-05
miR-6089-2	2.83	2.72E-07	1.53E-05
miR-4690-5p	2.82	2.15E-05	0.0005
miR-224-5p	2.79	0.0001	0.0023
miR-1915-3p	2.78	4.34E-08	5.64E-06
miR-6068	2.76	3.69E-05	0.0008
miR-6813-5p	2.7	2.36E-05	0.0005
miR-4539	2.7	1.55E-05	0.0004
miR-7108-5p	2.69	3.68E-08	5.12E-06
miR-6726-5p	2.67	0.0043	0.0474

miR-4695-5p	2.65	2.34E-05	0.0005
miR-4758	2.63	0.0023	0.0277
miR-4655-5p	2.62	9.05E-05	0.0017
miR-4758	2.61	0.0053	0.0563
miR-3131	2.6	3.93E-05	0.0008
miR-4685-5p	2.58	0.0004	0.0065
miR-92b-5p	2.58	4.13E-05	0.0008
miR-3656	2.57	4.11E-07	2.14E-05
miR-1207-5p	2.56	4.50E-06	0.0001
miR-933	2.52	9.82E-06	0.0003
miR-4651	2.52	3.14E-06	0.0001
miR-1908-5p	2.51	5.41E-06	0.0002
miR-6724-5p	2.51	9.66E-08	8.11E-06
miR-6510-5p	2.51	4.22E-05	0.0009
miR-1227-5p	2.5	7.97E-06	0.0002
miR-6727-5p	2.5	6.30E-07	3.03E-05
miR-6858-5p	2.49	5.40E-07	2.61E-05
miR-6125	2.49	1.20E-07	9.35E-06
miR-3185	2.46	5.13E-07	2.51E-05
miR-6821-5p	2.45	1.10E-06	4.65E-05
miR-2861	2.45	1.46E-07	1.08E-05
miR-3656	2.45	8.11E-06	0.0002
miR-4466	2.44	4.16E-07	2.16E-05
miR-5572	2.43	0.0015	0.0193
miR-6088	2.43	1.77E-07	1.19E-05
miR-5787	2.43	3.62E-06	0.0001
miR-4530	2.39	1.91E-07	1.26E-05
miR-6743-5p	2.38	1.64E-05	0.0004
miR-8073	2.37	1.13E-06	4.70E-05
miR-6087	2.37	1.13E-06	4.70E-05
miR-4646-5p	2.36	1.92E-05	0.0004
miR-6729-5p	2.35	1.61E-07	1.12E-05
miR-4466	2.35	2.47E-05	0.0005

miR-4707-5p	2.33	3.14E-07	1.73E-05
miR-4787-5p	2.32	3.02E-07	1.68E-05
miR-4674	2.31	0.0001	0.0027
miR-3154	2.3	3.12E-05	0.0007
miR-664b-5p	2.3	1.57E-05	0.0004
miR-6800	2.3	6.40E-06	0.0002
miR-6800	2.3	6.40E-06	0.0002
miR-4749-5p	2.3	0.0002	0.004
miR-6781-5p	2.29	0.0013	0.0172
miR-638	2.29	1.20E-06	4.96E-05
miR-762	2.29	1.13E-05	0.0003
miR-4763-3p	2.29	1.08E-06	4.61E-05
miR-4785	2.27	2.77E-06	9.43E-05
miR-6784-5p	2.27	2.20E-05	0.0005
miR-5095	2.25	0.0003	0.0044
miR-1237-5p	2.24	3.91E-05	0.0008
miR-5703	2.22	1.99E-07	1.28E-05
miR-5001-5p	2.22	1.21E-05	0.0003
miR-6090	2.22	0.0002	0.0033
miR-485-5p	2.21	0.0113	0.1023
miR-4270	2.18	6.68E-07	3.14E-05
miR-5571-5p	2.18	7.18E-05	0.0014
miR-6769b-5p	2.17	0.0025	0.0293
miR-4687-3p	2.14	2.24E-06	8.09E-05
miR-6741-5p	2.14	8.19E-06	0.0002
miR-6800-5p	2.12	1.89E-06	7.07E-05
miR-4430	2.1	1.53E-05	0.0004
miR-8064	2.09	0.031	0.2221
miR-4743-5p	2.08	0.0042	0.046
miR-8069	2.07	9.28E-07	4.07E-05
miR-1233-5p	2.07	0.0015	0.0184
miR-4508	2.07	1.36E-05	0.0003
miR-6789-5p	2.05	9.36E-06	0.0002

miR-3178	2.04	2.09E-06	7.70E-05
miR-324-5p	2.04	0.0009	0.013
miR-6860	2.04	0.0011	0.0149
miR-504-3p	2.03	0.0004	0.0055
miR-328-5p	2.02	0.0002	0.0028
miR-4732-5p	2.02	0.0097	0.0897
miR-7111-5p	2.02	0.0005	0.0075
miR-4485	2.02	0.0047	0.0504
miR-6090	2.01	8.11E-07	3.61E-05
