# Supplementary material for: Three-Dimensional Organotypic Cultures Reshape the microRNAs Transcriptional Program in Breast Cancer Cells
Source: Cancers (Basel). 2022 May 19;14(10):2490. doi: 10.3390/cancers14102490 (PMC9139376; doi:10.3390/cancers14102490)
Supplement: Supplementary file 1 [file cancers-14-02490-s001.zip › Supplementary Table S2.pdf]

Table S2. List of the 149 miRNAs downregulated in 3D cultures compared to 2D.

miRNA	Fold Change	p-value	FDR
miR-5189-3p	-26.31	6.61E-07	3.13E-05
miR-3160-1	-25.52	9.72E-09	2.15E-06
miR-183-5p	-21	1.03E-07	8.44E-06
miR-935	-19.58	8.67E-08	7.46E-06
miR-181a-2-3p	-18.72	2.62E-07	1.53E-05
miR-20a-5p	-18.66	1.80E-09	9.96E-07
miR-25-5p	-16.26	6.75E-08	6.78E-06
miR-615-3p	-15.57	4.96E-07	2.47E-05
miR-152-3p	-14.88	1.91E-08	3.52E-06
miR-140-3p	-13.77	5.83E-09	1.61E-06
miR-143-3p	-13.46	6.01E-10	5.70E-07
let-7g-5p	-12.55	4.14E-09	1.32E-06
miR-17-5p	-12.54	3.23E-09	1.26E-06
miR-3663-3p	-12.41	4.65E-06	0.0001
miR-28-5p	-12.38	2.60E-10	2.87E-07
miR-3160-1	-12.14	6.82E-05	0.0013
miR-1260b	-11.99	7.97E-08	7.08E-06
miR-106a-5p	-10.95	4.62E-11	1.02E-07
miR-22-3p	-10.35	7.29E-08	6.90E-06
miR-196a-5p	-10.25	2.87E-09	1.26E-06
miR-29b-1-5p	-9.81	3.19E-08	4.83E-06
miR-92b-3p	-9.76	8.34E-10	6.92E-07
miR-195-5p	-9.54	2.65E-06	9.15E-05
miR-629-5p	-9.07	3.89E-07	2.07E-05
miR-512-3p	-8.57	4.70E-07	2.40E-05
miR-138-5p	-8.37	4.25E-09	1.32E-06
miR-1323	-8.18	1.91E-07	1.26E-05
miR-3911	-8.08	7.01E-06	0.0002
miR-34c-3p	-7.85	0.0015	0.0192

miR-182-5p	-7.52	1.38E-09	8.32E-07
miR-151b	-7.3	1.49E-06	5.89E-05
miR-27b-5p	-7.14	3.61E-08	5.12E-06
miR-27b-3p	-7.07	6.37E-07	3.04E-05
miR-155-5p	-7.01	2.29E-06	8.21E-05
miR-151a-3p	-7	2.39E-07	1.43E-05
miR-181d-5p	-6.86	1.01E-05	0.0003
miR-28-3p	-6.3	5.41E-08	5.82E-06
miR-519c-5p	-6.22	2.60E-05	0.0006
miR-519b-5p	-6.22	2.60E-05	0.0006
miR-523-5p	-6.22	2.60E-05	0.0006
miR-518e-5p	-6.22	2.60E-05	0.0006
miR-522-5p	-6.22	2.60E-05	0.0006
miR-519a-5p	-6.22	2.60E-05	0.0006
miR-1180-3p	-6.14	3.75E-06	0.0001
miR-378a-3p	-5.97	1.58E-08	3.18E-06
miR-125b-1-3p	-5.92	1.15E-05	0.0003
miR-138-1-3p	-5.83	2.22E-07	1.35E-05
miR-92b	-5.82	2.27E-09	1.16E-06
miR-146a-5p	-5.82	2.71E-07	1.53E-05
miR-5100	-5.76	7.77E-09	1.89E-06
miR-23b-5p	-5.65	1.11E-06	4.65E-05
hsa-let-7f-5p	-5.63	6.00E-06	0.0002
miR-99b-3p	-5.6	3.75E-06	0.0001
miR-4669	-5.36	2.69E-07	1.53E-05
miR-330-3p	-5.27	1.25E-07	9.56E-06
miR-16-5p	-5.25	3.75E-09	1.32E-06
miR-181b-5p	-5.14	1.37E-09	8.32E-07
miR-526b-5p	-5.13	5.17E-05	0.001
miR-517a-3p	-4.92	2.63E-06	9.15E-05
miR-517b-3p	-4.92	2.63E-06	9.15E-05

miR-185-5p	-4.91	4.24E-09	1.32E-06
miR-652-3p	-4.91	7.07E-06	0.0002
miR-130a-3p	-4.9	4.93E-07	2.47E-05
miR-7977	-4.88	3.04E-06	0.0001
miR-130b-3p	-4.74	5.25E-08	5.82E-06
miR-23a-5p	-4.63	1.13E-08	2.41E-06
miR-26a-5p	-4.6	1.74E-08	3.30E-06
miR-378c	-4.58	1.90E-05	0.0004
miR-125a-5p	-4.53	2.20E-08	3.94E-06
miR-505-5p	-4.4	0.0033	0.0371
miR-6831-5p	-4.38	1.03E-07	8.44E-06
miR-484	-4.26	1.49E-07	1.08E-05
miR-107	-4.23	3.24E-09	1.26E-06
miR-103a-3p	-4.03	1.73E-08	3.30E-06
miR-342-3p	-4.03	4.63E-08	5.64E-06
miR-7162-3p	-3.98	5.15E-07	2.51E-05
miR-197-3p	-3.93	2.83E-06	9.57E-05
miR-296-3p	-3.9	2.98E-06	9.98E-05
miR-339-5p	-3.74	0.0002	0.003
miR-191-5p	-3.7	2.75E-08	4.68E-06
miR-671-3p	-3.65	0.0003	0.0053
miR-21-3p	-3.62	7.75E-06	0.0002
miR-4298	-3.47	7.62E-08	7.08E-06
miR-498	-3.46	1.10E-06	4.65E-05
miR-214-3p	-3.43	3.12E-08	4.83E-06
miR-484	-3.34	1.98E-05	0.0005
miR-1270	-3.33	0.0044	0.0474
miR-23b-3p	-3.31	1.23E-08	2.55E-06
miR-425-5p	-3.29	6.79E-07	3.17E-05
miR-516b-5p	-3.28	2.45E-05	0.0005
miR-151a-5p	-3.28	4.46E-08	5.64E-06

miR-4726-5p	-3.27	1.35E-06	5.48E-05
miR-125a-3p	-3.12	2.52E-05	0.0005
hsa-let-7d-5p	-3.09	8.01E-08	7.08E-06
miR-487b-3p	-3	0.0011	0.015
miR-7847-3p	-3	8.12E-08	7.09E-06
miR-767-5p	-2.99	2.07E-05	0.0005
miR-93-5p	-2.94	2.08E-07	1.31E-05
miR-485-3p	-2.93	9.38E-08	7.97E-06
miR-92a-1-5p	-2.93	9.05E-05	0.0017
miR-1307-3p	-2.92	9.75E-07	4.23E-05
miR-106b-5p	-2.92	3.98E-05	0.0008
miR-193a-5p	-2.91	1.24E-07	9.56E-06
miR-423-3p	-2.9	3.70E-07	1.99E-05
miR-105-5p	-2.9	0.0076	0.0738
miR-584-5p	-2.88	0.0023	0.0276
miR-199a-3p	-2.86	4.82E-06	0.0001
miR-199b-3p	-2.86	4.82E-06	0.0001
miR-504-5p	-2.86	9.49E-07	4.14E-05
miR-532-3p	-2.84	0.0008	0.0114
miR-574-3p	-2.81	1.94E-06	7.24E-05
miR-520d-3p	-2.79	3.39E-05	0.0007
miR-378f	-2.78	0.0044	0.0477
miR-15b-5p	-2.77	2.18E-07	1.34E-05
miR-379-5p	-2.74	7.17E-05	0.0014
miR-328-3p	-2.73	0.0006	0.0084
miR-24-3p	-2.72	5.44E-08	5.82E-06
hsa-let-7e-5p	-2.65	4.09E-08	5.43E-06
miR-181a-5p	-2.65	3.42E-07	1.87E-05
miR-422a	-2.65	0.0004	0.0055
miR-2110	-2.64	0.0208	0.1666
miR-4429	-2.63	2.57E-07	1.52E-05

miR-145-5p	-2.63	1.10E-07	8.88E-06
miR-486-5p	-2.62	4.50E-05	0.0009
miR-30a-3p	-2.6	2.69E-06	9.24E-05
miR-331-3p	-2.59	1.47E-05	0.0004
miR-181c-5p	-2.56	0.0034	0.0387
miR-6808-3p	-2.53	0.0079	0.077
miR-27a-5p	-2.52	4.78E-05	0.001
miR-526a	-2.46	0.0002	0.0034
miR-520c-5p	-2.46	0.0002	0.0034
miR-518d-5p	-2.46	0.0002	0.0034
miR-23a-3p	-2.4	1.94E-07	1.26E-05
miR-4443	-2.38	6.34E-06	0.0002
miR-455-3p	-2.36	1.72E-06	6.56E-05
miR-320e	-2.34	9.64E-06	0.0002
miR-361-5p	-2.34	7.55E-06	0.0002
miR-106b-3p	-2.34	0.0003	0.0042
hsa-let-7a-5p	-2.28	1.65E-06	6.33E-05
miR-744-5p	-2.28	6.09E-06	0.0002
miR-1271-5p	-2.25	0.0032	0.0371
miR-1301-3p	-2.24	0.0009	0.012
miR-27a-3p	-2.21	1.30E-05	0.0003
miR-4454	-2.16	3.79E-07	2.03E-05
miR-365b-5p	-2.11	0.0006	0.0093
miR-4284	-2.06	0.0081	0.078
miR-891a-5p	-2.03	0.0005	0.0079
miR-2115-5p	-2.01	0.0044	0.048
miR-3195	-2	3.90E-06	0.0001
