# Supplementary figures and images for: Three-Dimensional Organotypic Cultures Reshape the microRNAs Transcriptional Program in Breast Cancer Cells
Source: Cancers (Basel). 2022 May 19;14(10):2490. doi: 10.3390/cancers14102490 (PMC9139376; doi:10.3390/cancers14102490)

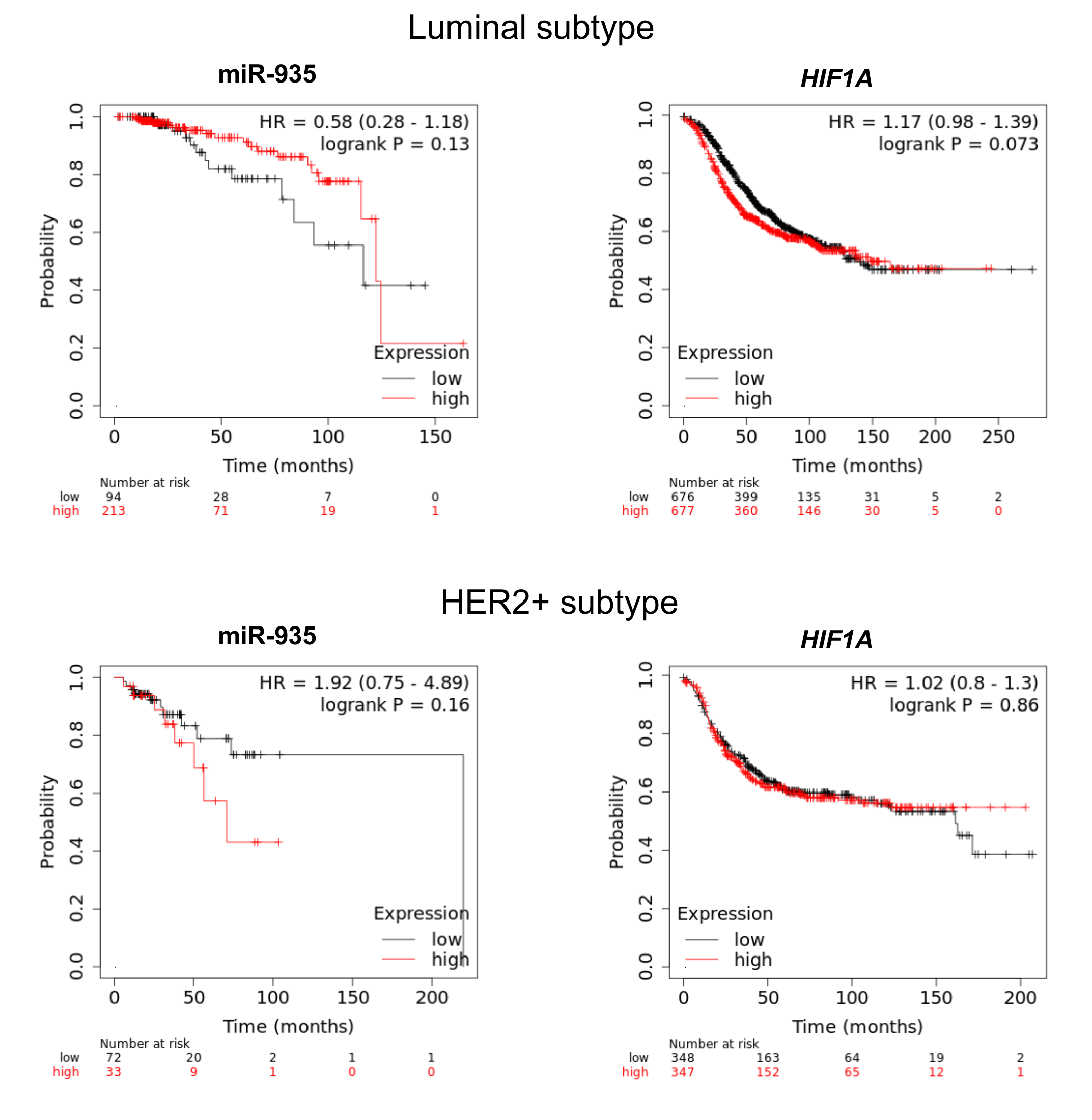

Supplement: Supplementary file 1 [file cancers-14-02490-s001.zip › Suplementary Figure S1.tif]
